# Single-cell expression quantitative trait loci (eQTL) analysis of SLE-risk loci in lupus patient monocytes

**DOI:** 10.1186/s13075-021-02660-2

**Published:** 2021-11-30

**Authors:** Yogita Ghodke-Puranik, Zhongbo Jin, Kip D. Zimmerman, Hannah C. Ainsworth, Wei Fan, Mark A. Jensen, Jessica M. Dorschner, Danielle M. Vsetecka, Shreyasee Amin, Ashima Makol, Floranne Ernste, Thomas Osborn, Kevin Moder, Vaidehi Chowdhary, Carl D. Langefeld, Timothy B. Niewold

**Affiliations:** 1 Colton Center for Autoimmunity, NYU Grossman School of Medicine, 550 1st Ave, New York, NY 10016 USA; 2 Department of Pathology, Immunology and Laboratory Medicine, University of Florida, Gainesville, FL USA; 3 Department of Biostatistics and Data Science and Center for Precision Medicine, Wake Forest School of Medicine, Winston-Salem, NC USA; 4 Department of Rheumatology, Ren Ji Hospital, School of Medicine, Shanghai Jiao Tong University, Shanghai, China; 5 Department of Immunology and Division of Rheumatology, Mayo Clinic, Rochester, MN USA; 6 Division of Rheumatology, Mayo Clinic, Rochester, MN USA; 7 Division of Rheumatology, Allergy and Immunology, Yale University School of Medicine, New Haven, USA

## Abstract

**Background:**

We performed expression quantitative trait locus (eQTL) analysis in single classical (CL) and non-classical (NCL) monocytes from patients with systemic lupus erythematosus (SLE) to quantify the impact of well-established genetic risk alleles on transcription at single-cell resolution.

**Methods:**

Single-cell gene expression was quantified using qPCR in purified monocyte subpopulations (CD14^++^CD16^−^ CL and CD14^dim^CD16^+^ NCL) from SLE patients. Novel analysis methods were used to control for the within-person correlations observed, and eQTLs were compared between cell types and risk alleles.

**Results:**

The SLE-risk alleles demonstrated significantly more eQTLs in NCLs as compared to CLs (*p* = 0.0004). There were 18 eQTLs exclusive to NCL cells, 5 eQTLs exclusive to CL cells, and only one shared eQTL, supporting large differences in the impact of the risk alleles between these monocyte subsets. The *SPP1* and *TNFAIP3* loci were associated with the greatest number of transcripts. Patterns of shared influence in which different SNPs impacted the same transcript also differed between monocyte subsets, with greater evidence for synergy in NCL cells. *IRF1* expression demonstrated an on/off pattern, in which expression was zero in all of the monocytes studied from some individuals, and this pattern was associated with a number of SLE risk alleles. We observed corroborating evidence of this *IRF1* expression pattern in public data sets.

**Conclusions:**

We document multiple SLE-risk allele eQTLs in single monocytes which differ greatly between CL and NCL subsets. These data support the importance of the *SPP1* and *TNFAIP3* risk variants and the IRF1 transcript in SLE patient monocyte function.

**Supplementary Information:**

The online version contains supplementary material available at 10.1186/s13075-021-02660-2.

## Introduction

Systemic lupus erythematosus (SLE) is a poorly understood autoimmune syndrome driven by the interplay of genetic and environmental influences, which lead to a break in immunologic self-tolerance. Genetic studies in SLE have been successful in identifying more than 100 SLE susceptibility loci [1, 2]. Most of the genetic polymorphisms associated with SLE are not coding-change variants [3, 4]. They are either located in non-coding regulatory regions near the 5′ and 3′ regions of genes, in DNAse hyper-sensitivity sites, or are in perfect LD with DNAse hypersensitivity sites. This suggests modulation of transcription as a likely mechanism by which many SLE-risk loci impact immune system biology [2], and data from many complex diseases support this idea [5]. Importantly, there is substantial variation in the pattern of DNAse hyper-sensitivity among different human cell types, supporting the idea that polymorphisms can tune gene expression in a highly cell-specific manner [5]. Thus, examining multiple cell types will be critical in determining the function of SLE-risk loci, as it is likely that the regulatory influence of these polymorphisms vary across cell types.

Transcriptomic studies in SLE using whole blood, peripheral mononuclear cells, or whole tissue are confounded by variations in the numbers and types of cells found within different samples and between individuals. In such studies, the relative proportion of contributing cell subsets can influence gene expression profile based on the unique gene signature related to their functions [6, 7], making it more difficult to interpret the biological significance of the observed differential gene expression. For example, it is impossible to determine if the difference in gene expression is shared homogeneously in all cells, or if the observed difference in gene expression is primarily driven by divergent gene expression in one particular cell subset, or if the difference arises solely due to a difference in proportions of specific cell types [8]. Similarly, an impact of the risk locus on gene expression in a minor cell subset may not be observed within a bulk cell data set. The situation could be even more complex, as each of these possibilities could be present in varying proportional degrees across samples within a given study. While de-convolution methods can be used, it is easy to envision scenarios in which de-convolution would be of limited use (e.g., the same transcript is simultaneously up- and down-regulated in different cell types, to varying degrees) [9, 10]. An additional strength of single-cell gene expression studies is that correlations between transcripts represent within-cell correlations, while co-expression in mixed cell bulk samples could represent some within cell correlations, but also could be the result of complex relationships between cells of different types.

While most of the confirmed SLE-risk loci are located in or near genes with immune system function, for the vast majority, we do not understand their impact on cell biology and immune responses nor their influence on various immune cell subsets. For risk loci near genes of unknown molecular function, it is difficult to identify the relevant biological pathway and cell type(s) when considering functional follow-up experiments. This is a major challenge in SLE genetics as many risk loci have been definitively implicated in SLE pathogenesis, but their molecular function is poorly understood [2]. When considering using gene expression data in eQTL studies, the above advantages of single-cell gene expression data from purified cell populations are intriguing and would suggest that single-cell expression studies would more accurately indicate the biological impact of risk loci. Our group and others have previously studied gene expression in sorted immune cell populations as well as at single-cell level in SLE patients and found striking between-individual differences in gene expression between immune cell subsets and within the same immune cell types [1, 7, 9]. In this study, we use single-cell gene expression data from two important SLE monocyte subsets and perform a single-cell eQTL analysis. We selected seven SNPs from six established SLE risk loci and 90 target genes for this analysis. We observed many eQTLs that met statistical significance after adjusting for the within-individual correlation by modeling the individual as a random effect in a linear model and applying multiple testing correction. These results demonstrate the efficiency of single-cell eQTL approach to effectively detect the biological impact of risk loci. The associated eQTL transcripts largely differed between the two closely related monocyte subsets, making the case that risk locus function differently depending upon cell type. We also observed a great deal of diversity in the transcript lists associated with each risk SNP.

## Methods

### Patients and samples

Whole blood samples from 15 Female SLE patients fulfilling the American College of Rheumatology criteria for the diagnosis of SLE [11, 12] and five age-sex matched healthy controls were procured from the Mayo Clinic, Rochester, MN. Exclusion criteria included pregnancy, active acute infection, chronic infection (e.g., hepatitis C, HIV, etc.), and current intravenous therapy (e.g., methylprednisolone or cyclophosphamide). The institutional review board approved the study and all patients provided informed consent. The patient data were used for all eQTL analyses, and the control data were only used in the comparison of IRF1 expression. The control set was too small to analyze separately for eQTLs, and combining patient and control cells together for eQTL analysis could result in confounding due to the expected differences in gene expression between patients and controls. Control data were only used in the IRF1 expression analysis.

### Purification of classical (CD14^++^CD16^−^) and non-classical (CD14^dim^CD16^+^) monocytes

As previously described [9], CD14^++^CD16^−^classical (CL) monocytes and CD14^dim^CD16^+^ non-classical (NCL) monocytes were isolated from peripheral blood and purified using magnetic separation. Briefly, CL monocytes were first purified by negative selection using a modified Human Pan-Monocyte Isolation protocol (Miltenyi) with addition of anti-CD16-biotin (Miltenyi) into the biotin-antibody cocktail. The purity was further increased using subsequent CD14 positive selection (Miltenyi). NCL monocytes were purified similarly with addition of anti-CD14-biotin (Miltenyi) to the antibody cocktail for negative selection followed by CD16 microbeads (Miltenyi) for positive selection. Flow cytometry analysis showed that of the CL and NCL populations obtained, each contained > 95% of each desired cell type (Supplemental Fig. 1).

### C1 single-cell capture

Single-cells from each bulk monocyte subset were isolated using Fluidigm C1 Single-Cell Auto Prep System. Purified CL monocytes were stained with Molecular Probes™ CellTracker™ Green CMFDA Dye (Life Technologies), while NCL monocytes were unstained before loading to C1 Single-Cell Auto Prep Array Integrated Fluidic Circuits (IFCs). CL and NCL monocytes were then sequentially loaded onto the C1 Integrated Fluidic Circuit (IFC). CL vs. NCL monocyte lineage of individual cells was determined by direct visualization using fluorescent microscopy, and at the same time, empty wells and wells that contained more than one cell were marked to exclude from later analysis. The IFCs were then examined using fluorescent microscopy, and the captured cells were identified as CL (stained) or NCL (not stained). Wells that contained more than one cell were also noted to exclude from later analysis. We captured 470 CL and 394 NCL cells from the SLE patients in total, averaging between 50 to 60 single cells per patient across both monocyte subsets, after excluding doublets and fragments. These results represent a 60% capture site efficiency.

### Single cell PCR gene expression

A total of 90-target genes, relevant to monocyte function, that included major cytokines and pathway proteins involved in inflammation were selected for pre-amplification in the IFCs using the Fluidigm C1 Single-Cell Auto Prep System according to the manufacturer’s protocol. qPCR-based gene expression assay of the target gene pre-amplified cDNAs were carried out using 96.96 IFCs on the BioMark HD System (Fluidigm) as described in the protocol. Raw data was analyzed using the Fluidigm Real-Time PCR Analysis software (v. 4.1.2) and quality check was performed by inspecting melt curves, amplification curves. A failure score was calculated for each cell as described previously [9, 13]. Cells with failure score (total CT value) greater than two standard deviations above the mean were excluded from downstream analysis. The limit of detection CT values was set at 28 [10]; CT values greater than or equal to 28 were considered non-detected and were assigned a value of zero for analysis. Gene expression values were calculated by subtracting the threshold cycle value for each gene for each cell from the number of cycles in the PCR reaction. In this way, higher numbers represent greater gene expression, and lower numbers indicate less expression.

### Genotyping

Seven lupus risk single nucleotide polymorphisms (SNPs) in six gene loci, IRF5, IRF7, ITGAM, PTPN22, SPP1, and TNFAIP3 were genotyped for eQTL analysis. We selected well-established lupus risk polymorphisms from the literature which we thought may have function in monocytes [2]. The polymorphisms studied were as follows: IRF5 (rs10488631), IRF7 (rs1061502), ITGAM (rs1143679, rs1143689), PTPN22 (rs2476601), SPP1 (rs9138), and TNFAIP3 (rs2230926). Genotyping was performed using PCR allelic discrimination assays on a BioMark HD System (Fluidigm). The observed genotype frequencies of the studied SNPs did not deviate significantly from Hardy–Weinberg equilibrium.

### Statistical analysis

For the initial univariate analysis, gene expression data was separated in to three genotype categories for each bi-allelic SNP for each patient (homozygous minor allele, heterozygous, and homozygous major allele). Data in CL and NCL populations were separately analyzed, using non-parametric analyses (Mann-Whitney *U*). Even when considering eQTL associations that surpassed a Bonferroni correction for the number of comparisons (*P* = 8 × 10^−5^), this was found to be too permissive with respect to type I error (Supplemental Fig. 2) [14]. This was due to distributional properties of the data that demonstrated patterns of normal expression mixed with varying degrees of dropout data and significant within-person correlation in transcript values. To deal with these properties, data was reanalyzed for eQTL associations utilizing four separate approaches [15]. The first approach used a tweedie mixed-effects model [16] to simultaneously account for the dropout and the person-specific heterogeneity. Gene expression was modeled as the outcome and genotypes were modeled as predictors along with a random effect for individual. The second approach used a logistic mixed-effects model [17], where all non-zero gene expression values were assigned as ones and modeled as a binary outcome to compare the proportion of genes turned “on” or “off” for each SNP. Genes where the average proportion turned “on” exceeded 98% were dropped. The third approach also computed a mixed-effects model with just the non-zero gene expression values, assuming an underlying Gaussian distribution. Lastly, the proportion of genes turned “on” or “off” was computed within each individual and a simple analysis of variance was computed where the proportion was modeled as the outcome and the genotype as the predictor. A Benjamini-Hochberg false discovery rate was used to control for multiple comparisons and results meeting an FDR < 0.1 were retained [18]. As shown in Table 1, the logistic and proportional models provided the strongest ability to detect eQTLs (12 and 9 eQTLs respectively), followed by Gaussian (3 eQTLs), and tweedie (1 eQTL) models. eQTL lists were compared among risk alleles and between cell types to understand the degree to which effects were shared between cells types and the degree to which SLE-risk loci coordinately regulated the same transcripts. eQTLs were considered shared if they met the significance cutoff in both monocyte subsets and were in the same direction of association. These patterns of sharing are represented using Venn diagrams.

**Table 1:** List of significant eQTL associations detected by the various statistical methods in classical and non-classical monocytes at < 0.1 FDR

Gene (SNP rsID)	Associated transcript	Method	Monocyte subset
*ITGAM* (rs1143679)	*TLR7*	Logistic	Classical
*ITGAM* (rs1143683)	*JAK1*	Logistic	Classical
*TNFAIP3* (rs2230926)	*IRF8*	Proportion	Classical
*SPP1* (rs9138)	*ARG1*	Logistic	Classical
SPP1 (rs9138)	*IRF1*	Logistic	Classical
*SPP1* (rs9138)	*IRF4*	Logistic	Classical
*IRF5* (rs10488631)	*IRF1*	Logistic	Non-classical
*IRF7* (rs1061502)	*IRF1*	Logistic	Non-classical
*ITGAM* (rs1143679)	*ARG1*	Gaussian	Non-classical
*ITGAM* (rs1143679)	*TCF4*	Logistic	Non-classical
*ITGAM* (rs1143683)	*IL1B*	Gaussian	Non-classical
*ITGAM* (rs1143683)	*TNFA*	Gaussian	Non-classical
*TNFAIP3* (rs2230926)	*CD274*	Logistic	Non-classical
*TNFAIP3* (rs2230926)	*FCER1G*	Proportion	Non-classical
*TNFAIP3* (rs2230926)	*IL7R*	Proportion	Non-classical
*TNFAIP3* (rs2230926)	*STAT1*	Proportion	Non-classical
*TNFAIP3* (rs2230926)	*STAT2*	Tweedie	Non-classical
*TNFAIP3* (rs2230926)	*TNFA*	Logistic	Non-classical
*TNFAIP3* (rs2230926)	*TYK2*	Proportion	Non-classical
*PTPN22* (rs2476601)	*IL5*	Logistic	Non-classical
*SPP1* (rs9138)	*IFIT5*	Proportion	Non-classical
*SPP1* (rs9138)	*IL1A*	Proportion	Non-classical
*SPP1* (rs9138)	*IRF1*	Logistic	Non-classical
*SPP1* (rs9138)	*TLR3*	Proportion	Non-classical
*SPP1* (rs9138)	*TYK2*	Proportion	Non-classical

Analyses to detect modules of gene co-expression in the single-cell data were completed in each cell type separately (CL and NCL). Using the intersection of genes (common across all individuals), we built a pairwise gene-by-gene correlation matrix for each individual and each cell type. Each correlation matrix was averaged into a single correlation matrix to find a mean correlation across all individuals while removing the inter-individual differences. The mean correlation was then used to compute eigenvectors and eigenvalues and build a principal component analysis. From there, each individual cell was projected on to that principal component space and observed for differences by individual. Gene sets were retained if the absolute value of the individual loadings associated with highly explanatory principal components were greater than 0.7.

## Results

### Unique eQTL associations between CL and NCL monocytes

**Fig. 1:**
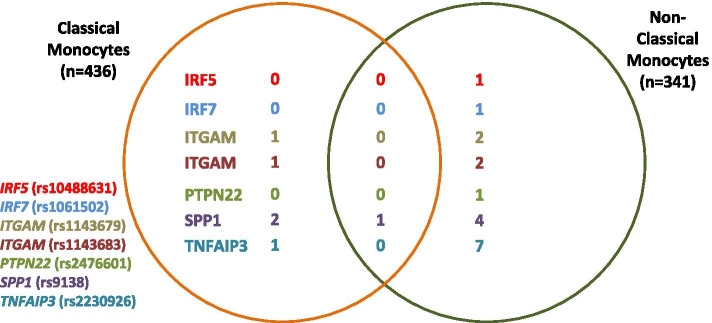
Venn diagram showing unique and shared eQTL associated transcripts between CL and NCL for each lupus risk SNP. Numbers indicate the number of transcripts associated with each SNP, with the numbers inside the overlap indicating transcript associations which are shared across the two monocyte subsets and those outside the overlap indicating unique SNP-transcript associations for each monocyte subset. The orange circle represents CL monocytes and the green circle represents NCL monocytes. Each lupus risk SNP is represented with different color

Using the four different analysis methods to query the data resulted in a total of 25 eQTL associations meeting a FDR < 0.1 (Table 1, Fig. 1). Interestingly, these largely differed between the two related monocyte subsets. There were 18 eQTLs exclusive to NCL cells, 5 eQTLs exclusive to CL cells, and one shared eQTL (Fig. 1, *p* = 0.0007 for a difference between the observed degree of sharing and a model in which 50% of eQTLs are shared between cell types). The SLE-associated SNPs demonstrated more eQTLs in NCLs compared to CLs (*p* = 0.0004). For a given SNP, the eQTL associated transcripts largely differed between cell types, with only one transcript-eQTL shared between CL and NCL cells (*SPP1* rs9138 with the IRF1 transcript). The greatest number of eQTLs was observed with the *SPP1* and *TNFAIP3* loci (7 and 8 eQTLs respectively). We included two missense SNPs in the *ITGAM* locus that have been shown evidence for independent biological function [19], and these two SNPs in the same locus were associated with different transcripts. These data indicate that the same risk allele had a different biological impact between the two monocyte subsets. This is striking given that the two monocyte subsets would largely be more closely related, than to B cells or T cells. These data suggest the importance of studying risk alleles within very specific cellular subsets to understand their biological roles. The different analysis methods used to detect eQTLs performed differently in the single-cell data, with logistic and proportional models detecting the greatest number of eQTLs (Table 1).

### Degree of eQTL transcript sharing between SLE-risk alleles

**Fig. 2:**
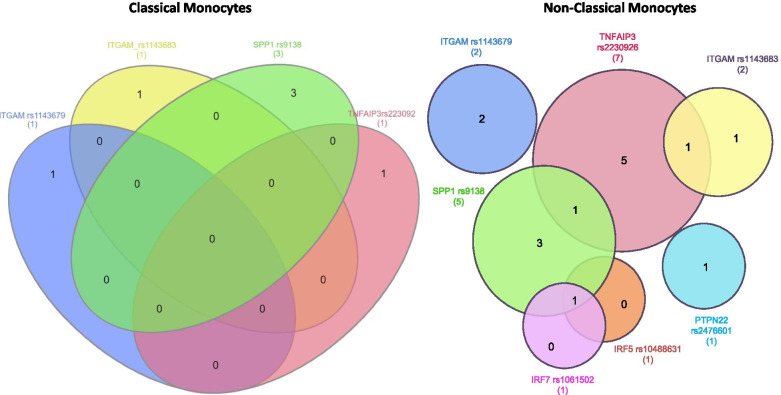
Comparison of eQTL lists for the different SLE-risk SNPs in two monocyte subsets. Venn diagram showing unique and shared eQTL transcripts associated with each risk allele for **A** CL and **B** NCL monocytes. The circles indicated by each color to represent one lupus risk SNP. Numbers in each area of the diagram represent the number of transcripts significantly associated with that risk allele, either separately or overlapping between risk alleles.

Next, we assessed whether different SNPs modulated the same transcripts (transcript sharing), as this could indicate different risk alleles converging on similar biological pathways. There were no transcripts shared among SNPs in CL cells (Fig. 2). In NCLs, two transcripts were common between two SNPs (*TNFA, TYK2*), and one transcript was common to three SNPs (*IRF1*). Interestingly, in the NCL cells SNPs in IRFs (*IRF5* and *IRF7*) are associated with *IRF1* expression. It is also notable that *IRF1* was the one eQTL that was shared between CL and NCL cells in the analyses above. Thus, while genetic variation in *IRF1* has not been associated with SLE, these analyses support the idea that *IRF1* expression is modulated by SLE genetic risk factors in monocyte lineage cells.

### On/off pattern of gene expression

**Fig. 3:**
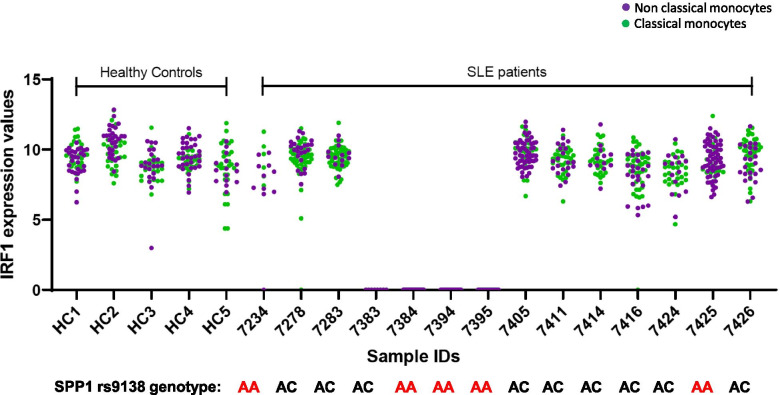
*IRF1* expression in CL and NCL monocytes in each individual separately. Gene expression values for *IRF1* are shown, with the cells from each individual in the study in a separate column. CL monocytes are shown in blue and NCLs in green, with each dot representing one cell. The genotypes under each column represent the *SPP1* rs9138 genotype in each person

Interestingly, the *IRF1* transcript demonstrated a highly binary expressed/not expressed pattern for all cells from a given individual, such that either all of the individual’s cells did not express the gene or the majority of cells showed *IRF1* expression (Fig. 3). This was true of both the CL and NCL monocytes from the same person. This pattern was restricted to patients and not observed in the controls in our study. We have seen this pattern in other single-cell qPCR studies of other diseases [20]. For example in a study of rheumatoid arthritis monocytes, we observed that *JAK1* expression fit this pattern, in patients only and not in controls [20]. *JAK1* did not fit this pattern in the present study of lupus patients, suggesting that this pattern of gene expression may be specific to the disease state. We searched public databases for other precedents of this on/off pattern of gene expression using Bio Turing browser version 2.5.3 [21]. We found a similar pattern for *IRF1* in monocytes from a single-cell RNA sequencing study examining patients with myeloma [22] (Supplemental Fig. 3). While our PCR data have a wider dynamic range of values than the public RNA-seq data, the on/off pattern appears similar between these two studies. This suggests that examining gene expression patterns in an individual is important, as this type of pattern is likely to be lost when individuals are pooled for analysis. The strength of the pattern in our data compared to RNA-seq data sets may indicate that these patterns are more efficiently detected in single cell qPCR data than in single cell RNA-seq data.

### Modular co-expression analysis of the single-cell data

**Fig. 4:**
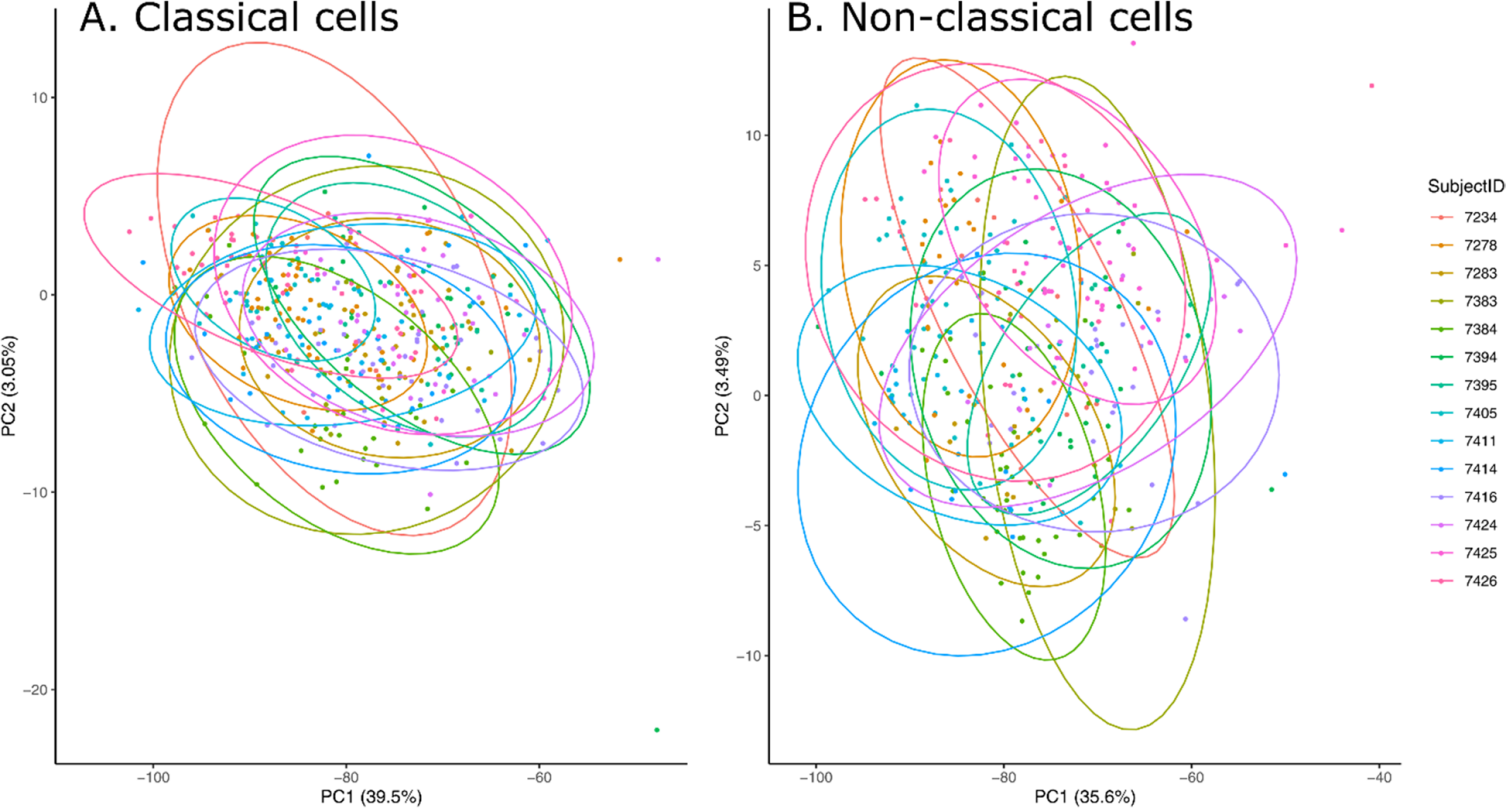
Principal component analyses of classical and non-classical cells. Each cell is a dot, and data are shown after adjusting for the inter-individual differences by averaging gene-gene correlation matrices across each individual and subsequently projecting cells onto to the principal component space. Cells are color-coded and circled by 95% confidence ellipses by subject identifiers. Large overlap demonstrates the removal of the individual-specific heterogeneity

The principal component analyses revealed much higher overlap of cells when correcting for inter-individual differences than not (Fig. 4). For classical cells, the first principal component explained 39.5% of the variance and the second principal component explained only 3.05% of the variance. Similarly, in non-classical cells, the first principal component explained 35.6% of the variance and the second principal component explained only 3.49% of the variance (Fig. 4). Thirty-two genes were associated with lower principal component 1 scores across both of the cell types (|loadings|> 0.7) (Table 2). Sixteen genes were associated with lower principal component 1 scores in non-classical cells (Table 2). Of those, 15 were shared in both cell types and only one (IFNG) was unique to NCLs, demonstrating a core set of co-expressed genes that are in common across both cell types (Table 2). In the CL cells, there were 16 additional genes that were co-expressed, supporting a larger co-expression network in this cell type.

**Table 2:** Co-expression networks, genes associated with lower principal component 1 scores (|loadings|> 0.7). These gene sets represent a set of co-expressed genes that explain the most variance in each dataset. A large portion of the genes are shared; however, classical cells demonstrate a much larger co-expression network

Classical	Shared	Non-classical
*CCR6*	*CCR2*	*IFNG*
*ITGAE*	*CCR5*	
*CD36*	*IDO1*	
*CD86*	*IFIH1*	
*FCER1G*	*IFIT3*	
*GMCSF*	*IL23A*	
*IFIT2*	*STAT3*	
*IFNB1*	*STAT5*	
*IL15*	*TLR3*	
*IL2*	*IL12B*	
*LILRA4*	*TRAF6*	
*PRDM1*	*FLT3*	
*STAT6*	*CTLA4*	
*TLR8*	*CXCR7*	
*TICAM1*	*CD80*	
*TYK2*		
*VCAN*		

## Discussion

In this study, we document a number of eQTLs associated with common autoimmune risk alleles for SLE in human monocytes, at a single-cell resolution. We studied patients, which may have increased our ability to detect eQTLs associated with these alleles, as the other requisite genetic background for SLE is also present in these individuals. The degree of difference in eQTL lists between monocyte subsets was striking, as these two cell types are more closely related to each other than other common immune cell types such as T cells and B cells. These data suggest that highly cell-type specific patterns of eQTLs are present in immune cells. Therefore, choosing the right cell types and including multiple cell types will be critical when studying risk alleles in immune mediated diseases. Screening of single-cell eQTL data [23] across multiple cell types would be an important strategy to decide upon which cell type to study in functional experiments, and our data support the limitations of gene annotation and presumed functions when considering the biological impact of the risk allele. One example of this would be the large number of trans associations we observe, which could not be predicted based upon the sequence location of the risk variant (e.g. SPP1(rs9138) associated with *IRF1* and *TYK2* transcripts).

It is interesting that we observed more eQTLs in the NCLs as compared to the CLs, as the cell numbers were similar between the two cell subsets and this is not related to statistical power. It could suggest that these risk alleles mediate their risk of disease to a greater degree via the NCL lineage as compared to the CL lineage. The structure of shared transcript modulation shown in Fig. 2 provides a map of the interactions between risk alleles at the biological level, and these data suggest greater coordination between risk alleles in NCL monocytes at least with respect to the variants and transcripts that we studied. Interestingly, while the number of eQTLs observed in CL cells was fewer, the co-expression network observed in this cell type contained a larger number of transcripts. This taken together with the analysis above would suggest that a fewer number of risk alleles are operative in CL cells but that these alleles result in a larger number of co-expressed transcripts. This finding should be tested in an RNA-seq experiment, as this conclusion is limited by the fact that we tested a prescribed set of transcripts in this study. Our data also support the overall importance of the *SPP1* and *TNFAIP3* risk alleles with respect to gene transcription in both CL and NCL monocytes. These data support the idea that different risk alleles will have their greatest effects in specific cell types, which will not be predictable from the magnitude of the effect size in case-control genetic association. The *SPP1* risk variant has been linked to innate immune system cytokine production in SLE previously [24], while *TNFAIP3* variants have been associated with differential TNFAIP3 function in monocyte lineage cells [25].

The on-off pattern of gene expression observed with *IRF1* is striking, and in comparison with public RNA-seq data sets it seems that the qPCR approach we have used illustrates this pattern more dramatically. This could be due to the more quantitative nature of PCR vs. shotgun sequencing. Biologically, this could relate to a strong transcriptional repressor, and it is interesting that we have observed this phenomenon in disease but not in controls, and in multiple disease states and with different transcripts [20]. This could indicate that the on/off gene expression pattern is related to either medication or to the underlying disease process. In our study, the *IRF1* transcript which was expressed in an on/off pattern was an eQTL. This could suggest genetic variation as a cause of the on/off pattern, although it is a trans-eQTL and thus would not represent a simple impact upon a *cis*-regulatory element. We have observed trans-eQTLs in this study despite measuring some of the transcripts for the annotated *cis*-gene variants being studied. We did not include each transcript in the region of the SNPs studied, and thus, we did not emphasize *cis*-eQTLs, but instead focused on monocyte-relevant transcripts that result from pathway activation events in the cell.

There are some limitations of this study. We have studied limited number of target genes and well-established SLE risk alleles; however, future studies are needed to include additional risk alleles and more diverse transcripts related to SLE pathogenesis. This will help in identifying additional eQTLs and in delineating the effect of risk variants in different cell types through *cis* or *trans* transcript regulation. Second, it is will be interesting to follow up the surprising on/off gene expression pattern in other disease states, larger control samples, and across different cell types. We expect that this should be done using single cell qPCR along with single cell RNA-seq, and the qPCR method may be more sensitive to detect this pattern.

## Conclusions

Studying single-cell eQTLs in SLE patient immune cells has allowed for novel insights which could not be achieved using previous mixed immune cell gene expression methods. These data support the importance of the *SPP1* and *TNFAIP3* risk variants and the IRF1 transcript in SLE patient monocyte function. This approach would be of great utility to detect differential transcription related to SLE-risk loci across multiple primary human cell types. This approach addresses a major frontier in complex autoimmune disease genetics, allowing us to understand how the function of a given risk allele varies by cell type in humans.

## Supplementary Information

**Additional file 1: Supplemental Figure 1.** Scatter plots of purified classical and non-classical monocytes. PBMCs = peripheral blood mononuclear cells, percentages in the gates for classical and non-classical monocytes indicate the purity of populations purified. **Supplemental Figure 2.** Quantile − quantile plot showing eQTL associations with SLE risk loci. A) Classical monocytes; B) Non-classical monocytes. A very early deviation of the observed from the expected P value for eQTL associations suggested high type I error, which we found to relate to within-person correlations and distributional properties of the single cell data as described in the paper. eQTL associations that remained significant after using the approaches described in the Methods to correct for the distributional properties of the data are highlighted in orange for classical and magenta for non-classical monocytes. **Supplemental Figure 3.** On/off pattern of IRF1 gene expression in monocytes from a single cell RNA sequencing study examining patients with multiple myeloma. X-axis shows individual patients and the Y-axis shows gene expression values for the IRF1 transcript. Each plotted dot represents the expression level for IRF1 in one cell. Bars show the median, error bars show the interquartile range. Data from public database as reported in Haradhvala, N.J., et al., Cancer Research, 2019.

## Data Availability

Primary data are available upon request to qualified investigators.

## Abbreviations

eQTL: Expression quantitative trait locus
CL: Classical
NCL: Non-classical
SLE: Systemic lupus erythematosus
IFN: Interferon
qPCR: Quantitative polymerase chain reaction
LD: Linkage disequilibrium
IFC: Integrated fluidic circuit
IRF1: Interferon regulatory factor 1
IRF5: Interferon regulatory factor 5
IRF7: Interferon regulatory factor 7
ITGAM: Integrin alpha M
PTPN22: Protein tyrosine phosphatase, non-receptor type 22
SPP1: Secreted phosphoprotein 1
TNFAIP3: Tumor necrosis factor alpha induced protein 3
