# Supplementary material for: Single-cell expression quantitative trait loci (eQTL) analysis of SLE-risk loci in lupus patient monocytes
Source: Arthritis Res Ther. 2021 Nov 30;23:290. doi: 10.1186/s13075-021-02660-2 (PMC8630910; doi:10.1186/s13075-021-02660-2)
Supplement: Supplementary file 1 — Additional file 1: Supplemental Figure 1. Scatter plots of purified classical and non-classical monocytes. PBMCs = peripheral blood mononuclear cells, percentages in the gates for classical and non-classical monocytes indicate the purity of populations purified. Supplemental Figure 2. Quantile − quantile plot showing eQTL associations with SLE risk loci. A) Classical monocytes; B) Non-classical monocytes. A very early deviation of the observed from the expected P value for eQTL associations suggested high type I error, which we found to relate to within-person correlations and distributional properties of the single cell data as described in the paper. eQTL associations that remained significant after using the approaches described in the Methods to correct for the distributional properties of the data are highlighted in orange for classical and magenta for non-classical monocytes. Supplemental Figure 3. On/off pattern of IRF1 gene expression in monocytes from a single cell RNA sequencing study examining patients with multiple myeloma. X-axis shows individual patients and the Y-axis shows gene expression values for the IRF1 transcript. Each plotted dot represents the expression level for IRF1 in one cell. Bars show the median, error bars show the interquartile range. Data from public database as reported in Haradhvala, N.J., et al., Cancer Research, 2019. [file 13075_2021_2660_MOESM1_ESM.pdf]

Supplemental figure 1

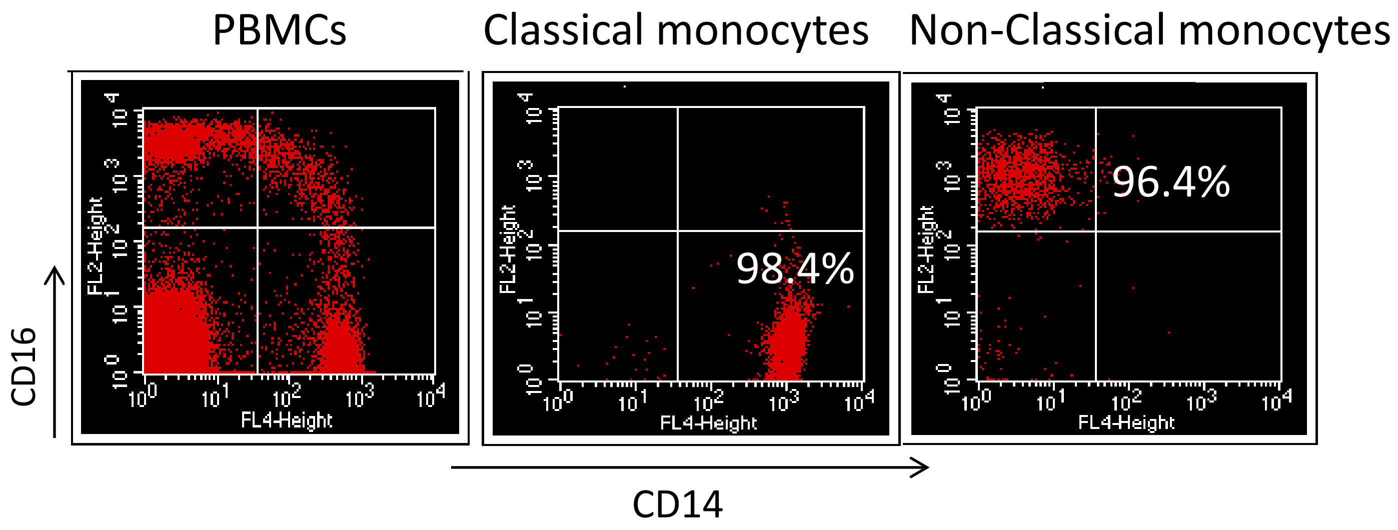

Scatter plots of purified classical and non-classical monocytes. PBMCs = peripheral blood mononuclear cells, percentages in the gates for classical and non-classical monocytes indicate the purity of populations purified

Supplemental Figure 2

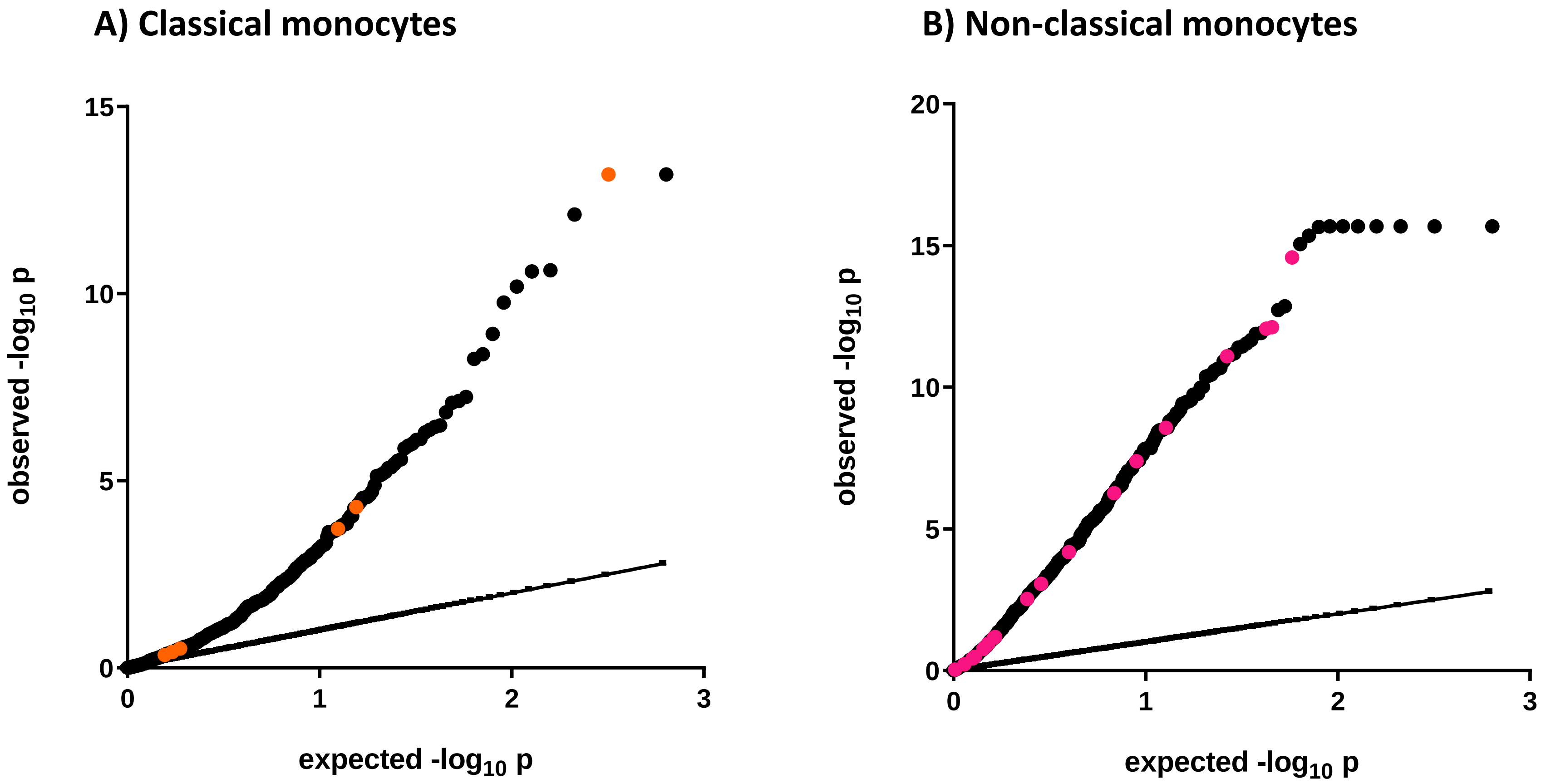

Quantile − quantile plot showing eQTL associations with SLE risk loci. A) Classical monocytes; B) Non-classical monocytes. A very early deviation of the observed from the expected P value for eQTL associations suggested high type I error, which we found to relate to within-person correlations and distributional properties of the single cell data as described in the paper. eQTL associations that remained significant after using the approaches described in the Methods to correct for the distributional properties of the data are highlighted in orange for classical and magenta for non-classical monocytes.

Supplemental Figure 3

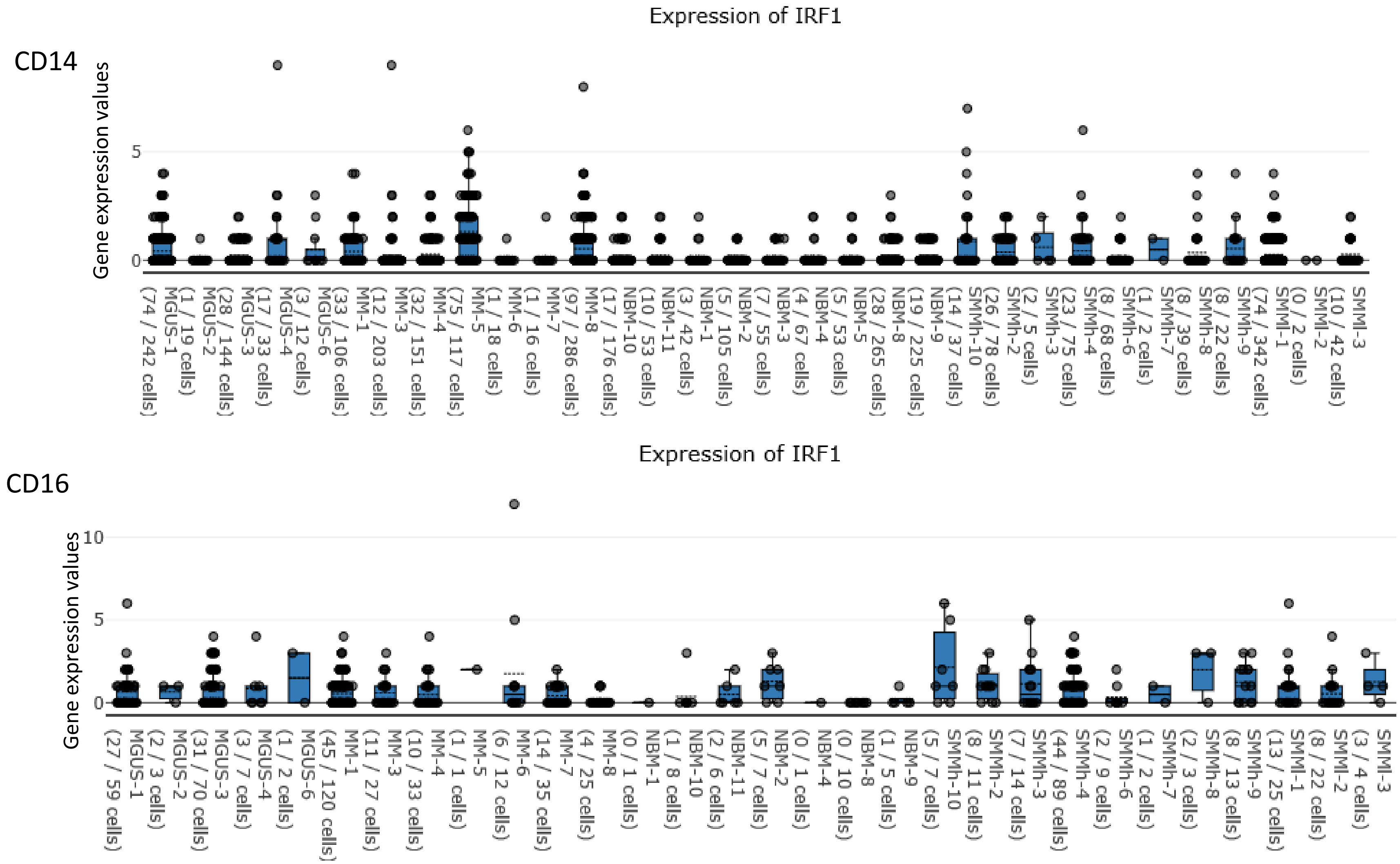

On/off pattern of IRF1 gene expression in monocytes from a single cell RNA sequencing study examining patients with multiple myeloma. X-axis shows individual patients and the Y-axis shows gene expression values for the IRF1 transcript. Each plotted dot represents the expression level for IRF1 in one cell. Bars show the median, error bars show the interquartile range. Data from public database as reported in Haradhvala, N.J., et al., Cancer Research, 2019.
